# Effects of Loading Duration and Short Rest Insertion on Cancellous and Cortical Bone Adaptation in the Mouse Tibia

**DOI:** 10.1371/journal.pone.0169519

**Published:** 2017-01-11

**Authors:** Haisheng Yang, Rachel E. Embry, Russell P. Main

**Affiliations:** 1 Musculoskeletal Biology and Mechanics Lab, Department of Basic Medical Sciences, Purdue University, West Lafayette, Indiana, United States of America; 2 Weldon School of Biomedical Engineering, Purdue University, West Lafayette, Indiana, United States of America; University of Notre Dame, UNITED STATES

## Abstract

The skeleton’s osteogenic response to mechanical loading can be affected by loading duration and rest insertion during a series of loading events. Prior animal loading studies have shown that the cortical bone response saturates quickly and short rest insertions between load cycles can enhance cortical bone formation. However, it remains unknown how loading duration and short rest insertion affect load-induced osteogenesis in the mouse tibial compressive loading model, and particularly in cancellous bone. To address this issue, we applied cyclic loading (-9 N peak load; 4 Hz) to the tibiae of three groups of 16 week-old female C57BL/6 mice for two weeks, with a different number of continuous load cycles applied daily to each group (36, 216 and 1200). A fourth group was loaded under 216 daily load cycles with a 10 s rest insertion after every fourth cycle. We found that as few as 36 load cycles per day were able to induce osteogenic responses in both cancellous and cortical bone. Furthermore, while cortical bone area and thickness continued to increase through 1200 cycles, the incremental increase in the osteogenic response decreased as load number increased, indicating a reduced benefit of the increasing number of load cycles. In the proximal metaphyseal cancellous bone, trabecular thickness increased with load up to 216 cycles. We also found that insertion of a 10 s rest between load cycles did not improve the osteogenic response of the cortical or cancellous tissues compared to continuous loading in this model given the age and sex of the mice and the loading parameters used here. These results suggest that relatively few load cycles (e.g. 36) are sufficient to induce osteogenic responses in both cortical and cancellous bone in the mouse tibial loading model. Mechanistic studies using the mouse tibial loading model to examine bone formation and skeletal mechanobiology could be accomplished with relatively few load cycles.

## Introduction

The skeleton is an adaptive structure that responds to mechanical loading by increasing bone mass under increased loads. The skeletal osteogenic response to externally applied mechanical loading is affected by various factors, two of which are loading duration (number of load cycles) and insertion of short-term rest intervals between load cycles [1–3]. Prior avian and rodent applied loading studies have shown that the cortical bone response to applied mechanical loading saturates after relatively few load cycles [2, 4–7]. Using the isolated avian ulna loading model to engender physiological strain magnitudes but with a non-physiological strain distribution, it was found that only 36 load cycles/day can produce an osteogenic response in cortical bone as effectively as 1800 cycles/day [4]. A similar study in rats trained to jump between 5 and 100 cycles/day showed that only 5 jumps/day were sufficient to induce a significant increase in cortical bone mass and bending stiffness, whereas 100 jumps/day only led to a modest increase in the cortical response compared to 40 jumps/day [6].

Short rests inserted between load cycles can also be important in enhancing mechanically induced bone formation in cortical bone [3, 8, 9]. Both the avian ulna axial-compression model and mouse tibia cantilever-bending model, have been used to demonstrate that insertion of 10 s rest periods following single load cycles transformed a low-magnitude, non-osteogenic loading regime into an osteogenic stimulus [3]. A related study using the mouse tibia cantilever-bending model also found that cortical bone formation was amplified by rest-insertion compared to continuous loading [10]. Similarly, in the rat tibia four-point bending model, load cycles interspersed with 14 s rest periods resulted in greater cortical bone formation rates compared to continuous load cycles, while rest periods less than 7 s did not enhance cortical osteogenesis [1].

Despite the insights gained for cortical bone through various animal loading models, the effects of loading duration and short rest insertion on the osteogenic response of cancellous bone to applied loading remain unknown. The in vivo mouse tibial loading model has been increasingly used for understanding the cellular mechanisms governing bone formation and the mechanobiological responses of bone tissue to mechanical loading simultaneously in cancellous and cortical bone tissues [11–13]. However, it remains unclear how varying loading duration and inserting short rest periods influence cancellous and cortical bone responses in this model, which induces a more physiological strain distribution throughout the tibia compared to prior avian and rodent models [14]. Furthermore, different mouse tibial loading studies have used loading regimes employing a variety of loading durations and rest insertion conditions [12, 13, 15–20], which makes it difficult to interpret the results of different studies relative to each other. Refinement of the number of loading protocols being used would be advantageous for enhancing comparability between studies from different groups as this loading model becomes increasingly used with transgenic animals to examine cellular mechanisms governing skeletal mechanobiology [21–24]. For example, different phenotypic responses to different loading protocols by the same transgenic model could heavily influence our interpretation of the role of certain proteins in skeletal mechanobiology and anabolic cellular pathways.

The objective of this study was to investigate the effects of loading duration and insertion of short rest intervals on the osteogenic responses of cancellous and cortical bone within the mouse tibia subjected to dynamic axial compressive loading. Based on prior observations for cortical bone from other animal loading models, we hypothesized that the cancellous and cortical osteogenic tissue response to loading would saturate after relatively few load cycles and a short periodic rest insertion (10 s) would improve the osteogenic response to loading.

## Materials and Methods

### Animals

Forty 16-week-old female C57BL/6 mice were used in the experiments. The mice were purchased from a commercial vendor (Taconic Biosciences, IN) and arrived at the age of 15 weeks at our animal facility. The mice were housed five per cage and allowed to acclimate in our animal facility for one week prior to the experiments. Water and rodent diet were provided ad libitum and a 12:12 light:dark cycle was maintained during the acclimation and experimental periods. The mice were weighed daily through the loading experiments as an indicator of health. All experimental procedures were approved by Purdue University’s Animal Care and Use Committee.

### Experimental design

**Fig 1:**
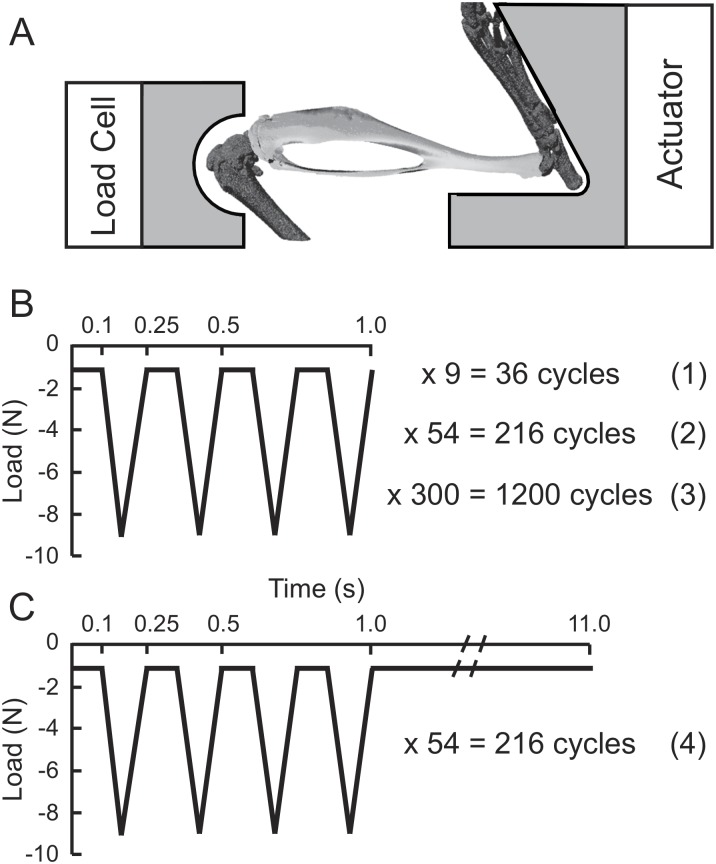
Schematics of the loading device (A) and loading signals (B and C). Four loading protocols: (1) 36 continuous load cycles/day; (2) 216 continuous load cycles/day; (3) 1200 continuous load cycles/day; (4) 216 cycles/day with 10 sec rest inserted between every 4 load cycles.

The forty mice were randomly divided into four groups (*n* = 10/group) and each group underwent two weeks of unilateral tibial loading (5 days/week, M-F) with each group undergoing a different loading protocol: (1) 36 continuous load cycles per day; (2) 216 continuous load cycles per day; (3) 1200 continuous load cycles per day; (4) 216 cycles per day with a 10 s rest inserted between every 4 load cycles (Fig 1). Protocols (1), (2) and (3) were used to examine the effect of loading duration (number of load cycles) on cortical and cancellous bone responses. Protocols (2) and (4) were used to determine whether insertion of a short rest interval between load cycles would augment the anabolic response. The number of load cycles (36, 216, 1200) and short rest interval (10 s) used here represented several typical values that have been commonly used in other in vivo loading studies [3, 4, 11, 17, 25, 26]. Choosing these previously used values facilitate direct comparisons between the current findings and others. Also, the number of load cycles (36, 216, 1200) tested here covers a relatively large range so that any load-induced responses in between these cycle numbers could be inferred. To examine only the effects of the number of load cycles and the rest insertion, all other loading parameters (e.g. load magnitude, load rate and waveform) were maintained the same for all four protocols (Fig 1).

### In vivo mechanical loading

In all four loading groups, cyclic compressive loads were applied to the left tibia of each mouse using a loading machine (Bose TestBench, TA Instruments, DE) fitted with custom fixtures to hold the hindlimb while the mouse was anesthetized [11, 17, 27, 28]. The right tibia served as a non-loaded control. The left tibia was maintained in the loading device using a -1 N pre-load. Compressive triangle waveform loads with -9 N peaks were applied at 4 Hz and characterized by 0.15 s of symmetric loading/unloading with a 0.10 s dwell (at -1 N) between load cycles [29] (Fig 1). The loading pattern used here was adopted from a well-established loading regime used in mouse tibial loading studies [11, 17, 26, 27]. Loading was applied at 4 Hz representing previous data reported for mouse stride frequency during running [30]. A previous study has shown that -1 N preload does not have any osteogenic effect on either cortical or cancellous bone of the tibia for this tibial loading model [17].

The peak load of -9 N was chosen because relevant tibial loading studies using this model have shown that -9 N is osteogenic for both the proximal metaphyseal cancellous bone and diaphyseal cortical bone of the tibiae of adult female C57BL6 mice (~16–20 week-old; [15, 20, 26, 27, 31]). In order to achieve the -9 N peak load received by the tibia, the actuator was commanded to achieve a slightly higher load value (-9.5 N) in the Wintest software associated with the loading machine and actual loads were recorded by the load cell and analyzed in Matlab (MathWorks, MA). The actual loads for 36-cycle, 216-cycle, 1200-cycle and 216-cycle with rest insertion were -9.03 N, -9.06 N, -9.01 N and -9.00 N, respectively. In protocol (4), a 10 s rest interval at -1 N followed every fourth load cycle (Fig 1), similar to a protocol used previously (5 s rest interval) in the mouse tibia, but with a longer rest interval here [17]. Previous studies using the avian ulna axial-compression model and the mouse tibia cantilever-bending model have shown that this rest interval (10 s) can improve cortical osteogenesis [3, 8, 10, 32]. Thus, we tested the 10 s rest interval to allow direct comparison with previous results. For an individual mouse, the daily loading periods for 36-cycle, 216-cycle, 1200-cycle and the rest-inserted 216-cycle loading protocols were 9 s, 1 min, 5 min and 10 min, respectively.

One mouse from the 36-cycle loading group experienced a tibial fracture on the fifth day of loading due to improper operation of the loading machine. The mouse was immediately euthanized and excluded from the study. All other mice tolerated the experiment well, as indicated by similar body mass in each experimental group before and after the two-week experimental period (*p* > 0.05, by paired *t*-test).

### Micro-computed tomography imaging and analyses

**Fig 2:**
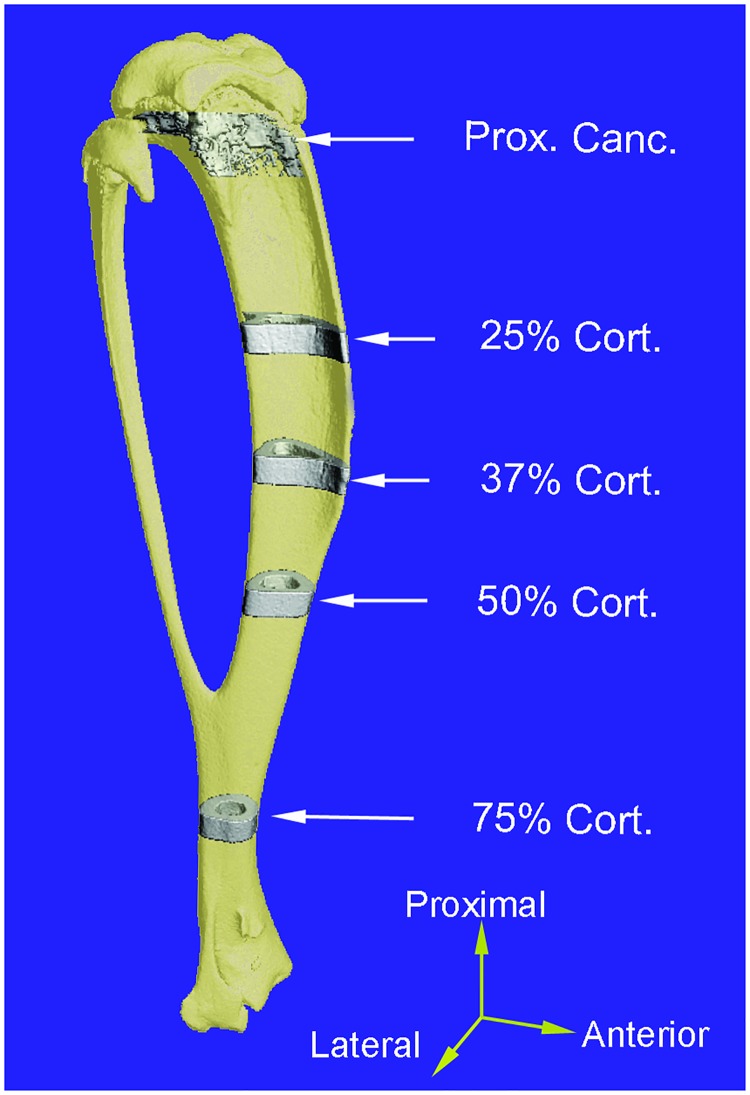
Locations of multiple diaphyseal cortical and metaphyseal cancellous volumes of interest (VOIs) used for microCT measurements. Cortical VOIs were centered at 25%, 37%, 50% and 75% of the tibial length relative to its proximal end, each extending 2.5% of the total tibial length. The cancellous VOI started just below the growth plate and extended 5% of the tibial length distally.

Following two weeks of loading, the mice were euthanized by carbon dioxide inhalation on day 15, three days after the last loading session. Intact tibiae were dissected from control and loaded limbs and scanned by micro-computed tomography (microCT) at an isotropic voxel size of 10 μm (μCT 40, Scanco Medical AG; 55 kVp, 145 mA, 300 ms integration time, no frame averaging) [28]. Prior to analysis, each tibia was aligned along its longitudinal axis using anatomical landmarks common to all mice [27]. Volumes of interest (VOIs) for proximal metaphyseal cancellous bone and diaphyseal cortical bone segments at distances of 25%, 37%, 50% and 75% of the bone’s length from its proximal end were defined in each tibia (Fig 2). The metaphyseal cancellous VOI began approximately 0.5 mm distal to the proximal growth plate, excluding the primary spongiosa and cortical shell, and extended 5% (~0.891 mm) of the total tibial length distally [17]. The metaphyseal cancellous bone and its surrounding cortex were separated by manually drawing contours around the cancellous bone volume, slice by slice, following the guidelines provided by Bouxsein et al [33] and this approach applied to all samples. Each of the diaphyseal cortical VOIs was centered at its respective position along the diaphysis and spanned 2.5% (~0.446 mm) of the total tibial length [11, 15]. The thresholds for segmenting cortical and cancellous bone tissues were determined separately according to the method used previously [27, 34] and confirmed by visual inspection. The same cortical and cancellous threshold values (372 mg HA/cm^3^ and 297 mg HA/cm^3^) were applied to the cortical and cancellous VOIs, respectively, across all control and loaded tibiae from the four loading groups.

Following bone segmentation, bone geometry and mineral density of the cortical and cancellous VOIs were measured using the scanner manufacturer’s software. The outcome parameters measured for the cortical VOIs included cortical bone area (Ct.Ar; mm^2^), total cross-sectional area (Tt.Ar; mm^2^), medullary area (Ma.Ar; mm^2^), cortical thickness (Ct.Th; mm), maximum and minimum principal moments of inertia (*I*_max_ and *I*_min_; mm^4^), and tissue mineral density (Ct.TMD; mg HA/cm^3^) as recommended [33]. The outcome parameters measured for the cancellous VOI included bone volume fraction (BV/TV; %), total volume (TV; mm^3^), trabecular thickness (Tb.Th; μm), trabecular number (Tb.N; 1/mm), trabecular separation (Tb.Sp; μm), and tissue mineral density (Cn.TMD; mg HA/cm^3^) as recommended [33].

### Statistical analyses

Two separate statistical analyses using linear mixed model with repeated measures [16, 27] were conducted to test for the interactive effects between different loading durations (36, 216 and 1200 daily load cycles) or for the effects of rest insertion (216 cycles with or without inserting a 10 s rest), where the within-subject factor was limb (control vs. loaded) and the between-subject factor was either the number of load cycles or the rest insertion (SPSS v 22.0, IBM, NY). All results presented are significant unless otherwise stated. Since we were primarily interested in the interactive effect of loading duration or short rest insertion on bone response to loading, if no significant interaction was present, only main effects were reported and no post-hoc pair-wise comparisons were performed. Where a significant interaction was found (*p* < 0.05), a post-hoc pair-wise comparison was conducted with a Bonferroni correction for repeated measures. All data are presented as mean ± SD. Percent differences between the loaded and control tibiae were calculated as: (loaded−control) ⁄control×100.

## Results

### Effect of load cycle number on cortical and cancellous bone response to mechanical loading

**Fig 3:**
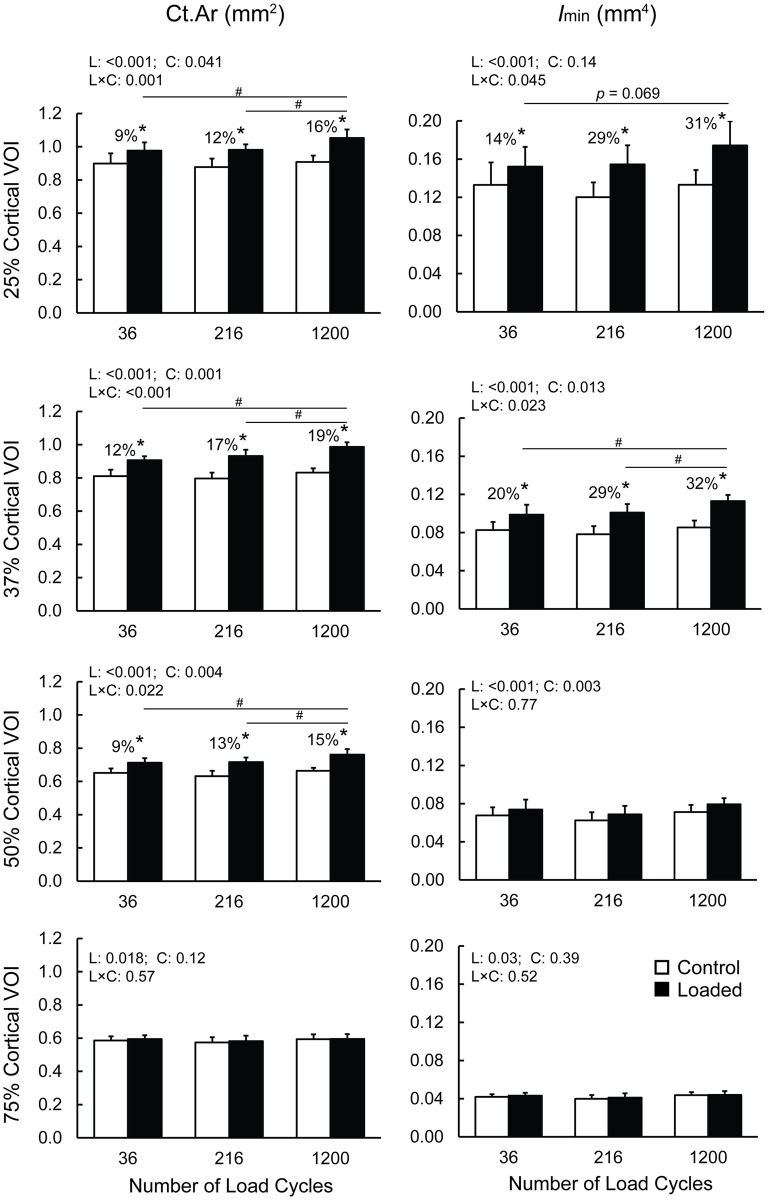
Effect of the number of load cycles (36, 216 and 1200) on cortical bone area (Ct.Ar) and minimum principal moment of inertia (*I*_min_) of the 25%, 37%, 50% and 75% cortical volumes of interest (VOIs). * indicates a significant difference between the control and loaded tibiae within the respective loading cycle group as determined by post-hoc pairwise comparisons when a statistical interaction is present indicating that the load-induced bone response is dependent upon the number of load cycles applied. # indicates a significant difference between the loaded tibiae for 1200 load cycles and 216 (or 36) load cycles while no difference exists between the nonloaded controls. P values indicating main effect of loading (L), main effect of load cycle number (C) and their interaction (L×C) are listed on top left of each plot. Percent differences were calculated as: (loaded−control) ⁄control×100.

**Fig 4:**
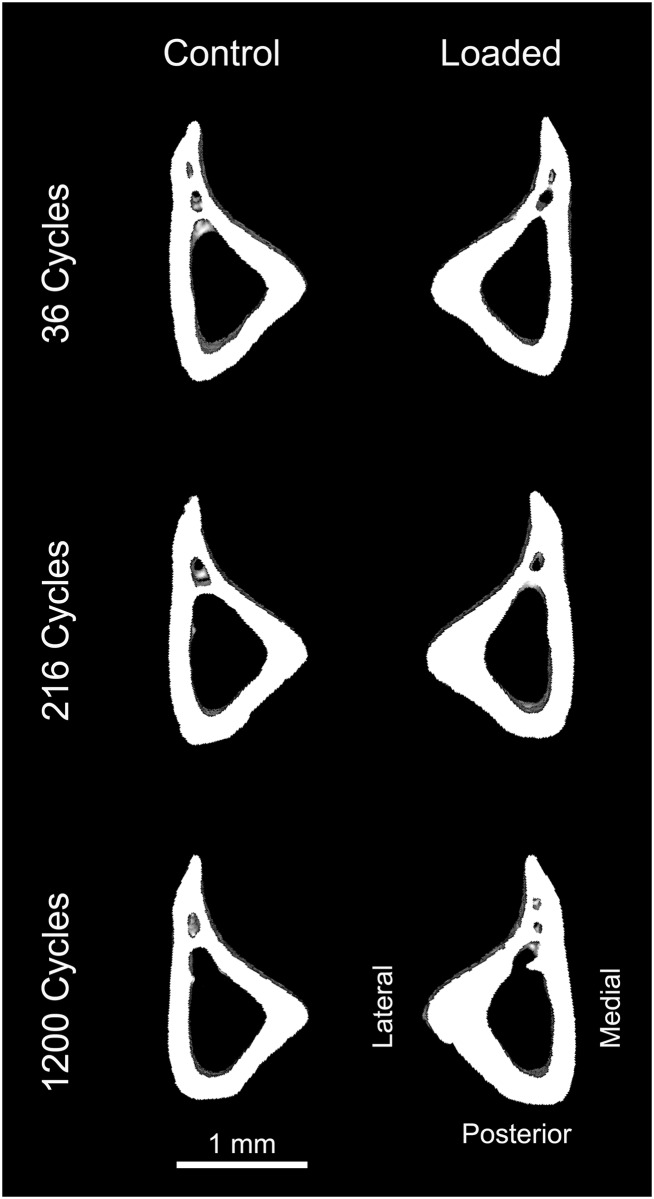
Representative microCT images of the 37% cortical volume of interest (VOI) in the control and loaded tibiae for 36, 216 and 1200 cycles of daily loading. The cortical area of the 37% VOI increased by 12%, 17% and 19% for 36, 216 and 1200 daily load cycles, respectively.

Daily applied dynamic loading consisting of 36, 216 and 1200 continuous load cycles all increased Ct.Ar in the cortical VOIs located 25%, 37% and 50% of the tibia’s length from its proximal end (Fig 3, S1 Table). These load-induced gains in Ct.Ar were greater for 1200 cycles than for 36 and 216 cycles. Applying 36 and 216 cycles induced similar increases in Ct.Ar (Fig 3, S1 Table). In the 25% cortical VOI, Ct.Ar was 9%, 12%, and 16% greater in the loaded relative to the control tibiae for 36, 216 and 1200 cycles, respectively. In the 37% cortical VOI, Ct.Ar was 12%, 17% and 19% greater in the loaded tibiae than the control tibae for 36, 216 and 1200 cycles, respectively (Fig 4). In the 50% cortical VOI, 36, 216 and 1200 cycles of loading induced increases in Ct.Ar of 9%, 13% and 15%, respectively. Loading had only a main effect on Ct.Ar at the 75% cortical VOI demonstrating only a small load-induced increase in Ct.Ar that was independent of the load cycle number (Fig 3, S1 Table). Similar results were observed for Ct.Th, except that in the 37% cortical VOI, loading increased Ct.Th more with both 216 and 1200 cycles than for 36 cycles (Table 1). In the 37% cortical VOI, *I*_min_ was 20%, 29% and 32% greater in the loaded tibiae relative to the control tibiae for 36, 216 and 1200 cycles, respectively (Fig 3, S1 Table). The increase in *I*_min_ with 1200 load cycles was greater than the osteogenic response for 36 or 216 load cycles (Fig 3, S1 Table). At the 25%, 50% and 75% VOIs, loading caused a significant increase in *I*_min_ that did not vary with load cycle number (S1 Table). A similar independence of load cycle number was found for *I*_max_ for the 25% and 37% VOIs and for Ct.TMD for the 37% VOI (Table 1). Loading led to increases in Tt.Ar at the 25%, 37% and 50% cortical VOIs as well as increases in Ma.Ar at the 25% and 37% VOIs, independent of load cycle number (Table 1).

**Table 1:** MicroCT measured parameters of diaphyseal cortical bone at distances of 25%, 37%, 50% and 75% of the tibial length from its proximal end and proximal metaphyseal cancellous bone, in mice subjected to axial compressive loading for 2 weeks under different daily load cycles (36, 216, 1200).

Parameters		36 Cycles		216 Cycles		1200 Cycles	
		Control	Loaded	Control	Loaded	Control	Loaded
Diaphyseal Cortical Bone							
Tt.Ar (mm^2^)	25% ^A^	3.804±0.299	3.952±0.211	3.584±0.218	3.904±0.273	3.768±0.155	3.943±0.243
	37% ^A^	3.101±0.151	3.319±0.164	2.994±0.181	3.227±0.196	3.135±0.191	3.366±0.218
	50% ^A^^,^^B^	1.582±0.073	1.650±0.105	1.508±0.099	1.609±0.091	1.632±0.059	1.725±0.068
	75%	1.194±0.043	1.209±0.050	1.153±0.069	1.199±0.128	1.196±0.040	1.197±0.049
Ma.Ar (mm^2^)	25% ^A^	2.905±0.243	2.976±0.172	2.706±0.185	2.923±0.259	2.860±0.145	2.891±0.210
	37% ^A^	2.291±0.123	2.412±0.147	2.197±0.157	2.296±0.169	2.303±0.172	2.379±0.212
	50% ^B^	0.931±0.052	0.937±0.081	0.877±0.075	0.893±0.070	0.968±0.050	0.963±0.068
	75%	0.608±0.036	0.614±0.035	0.580±0.051	0.617±0.138	0.603±0.028	0.601±0.025
Ct.Th (mm)	25% ^A^^,^^B^^,^^C^	**0.182±0.007**	**0.196±0.006**	**0.183±0.005**	**0.201±0.007**	**0.186±0.007**	**0.212±0.008** ^d^^,^^e^
	37% ^A^^,^^B^^,^^C^	**0.188±0.006**	**0.206±0.005**	**0.190±0.004**	**0.217±0.005** ^d^	**0.193±0.006**	**0.225±0.010** ^d^^,^^e^
	50% ^A^^,^^C^	**0.220±0.005**	**0.237±0.006**	**0.218±0.006**	**0.244±0.008**	**0.220±0.005**	**0.250±0.012** ^d^
	75%	0.237±0.010	0.239±0.004	0.235±0.009	0.239±0.009	0.238±0.009	0.237±0.009
*I*_max_ (mm^4^)	25% ^A^	0.297±0.034	0.323±0.027	0.281±0.034	0.315±0.035	0.298±0.019	0.337±0.028
	37% ^A^	0.232±0.024	0.261±0.020	0.217±0.021	0.257±0.028	0.241±0.019	0.276±0.028
	50% ^A^^,^^B^	0.079±0.06	0.093±0.010	0.074±0.009	0.092±0.009	0.083±0.005	0.103±0.006
	75%	0.055±0.004	0.056±0.005	0.051±0.005	0.051±0.005	0.055±0.005	0.056±0.005
Ct.TMD (mgHA/cm^3^)	25%	1030±7	1035±10	1033±6	1033±8	1039±8	1038±8
	37% ^A^	1047±8	1044±9	1050±9	1043±8	1052±8	1045±9
	50%	1105±12	1111±18	1113±9	1118±8	1115±9	1116±10
	75%	1100±11	1103±7	1101±9	1103±8	1116±9	1116±9
Metaphyseal Cancellous Bone							
TV (mm^3^)		1.87±0.14	1.88±0.16	1.83±0.14	1.87±0.15	1.88±0.14	1.87±0.19
Tb.N (1/mm)		3.5±0.2	3.4±0.2	3.6±0.2	3.6±0.3	3.5±0.3	3.5±0.2
Tb.Sp (μm)		283±21	285±19	274±20	273±25	280±26	280±24
Cn.TMD (mgHA/cm^3^)^A^		758±23	772±10	759±9	786±9	761±17	782±19

Data are given as mean ± SD.

^A^ main effect of loading;

^B^ main effect of load cycle number;

^C^ interactive effect of loading and load cycle number (within-subject factor: control vs. loaded, between-subject factor: number of load cycles applied).

^d^ different from the loaded tibiae for 36 cycles (no difference between nonloaded controls);

^e^ different from the loaded tibiae for 216 cycles (no difference between nonloaded controls).

Bold denotes a difference between the loaded and control tibiae within each load cycle group when an interaction is present.

**Fig 5:**
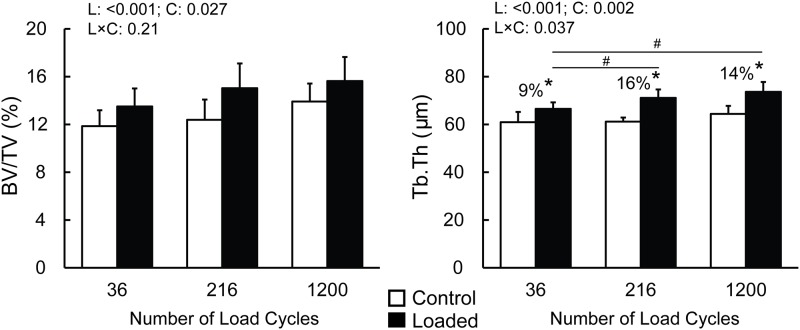
Effect of the number of load cycles on bone volume fraction (BV/TV) and trabecular thickness (Tb.Th) in the proximal metaphyseal canellous bone. * significant difference between the control and loaded tibiae within the respective loading cycle group, as determined by post-hoc pairwise comparisons when a statistical interaction is present indicating that the load-induced bone response is dependent upon the number of load cycles applied. # indicates a significant difference between the loaded tibiae for 36 load cycles and 216 (or 1200) load cycles, while no difference exists between the nonloaded controls. P values indicating main effect of loading (L), main effect of load cycle number (C) and their interaction (L×C) are listed on top left of each plot. Percent differences were calculated as: (loaded−control) ⁄control×100.

Cancellous BV/TV and Cn.TMD increased in response to applied loads. However, the load-induced changes in BV/TV and Cn.TMD were not statistically different between the three load durations examined (Fig 5, Table 1 and S1 Table). Cancellous TV was identical for control and loaded limbs across all load cycle groups and was unaffected by loading (Table 1). Tb.Th increased to a degree with increasing load cycle number where Tb.Th was 9%, 16% and 14% greater in the loaded relative to control tibiae for 36, 216 and 1200 load cycles, respectively (Fig 5). The load-induced increase in Tb.Th was lowest for 36 load cycles, but similar between 216 and 1200 load cycles (Fig 5, S1 Table). Tb.N and Tb.Sp were unaffected by the applied load for all three load durations tested (Table 1).

### Effect of rest insertion on the cortical and cancellous bone response to mechanical loading

Insertion of a 10 s rest after every fourth load cycle generally did not affect the magnitude of the load-induced response of the tibia to 216 applied load cycles. Loading with or without a rest insertion induced similar increases in Ct.Ar, Ct.Th and *I*_min_ in all cortical VOIs (25%, 37%, 50% and 75%) (Table 2). Similarly, loading generally increased *I*_max_ in the 25%, 37% and 50% cortical VOIs and Ct.TMD in the 37% cortical VOI (Table 2), but the load-induced increases in these measures were not affected by the presence of a rest-insertion. A significant interactive effect of the rest insertion was present for the 50% cortical VOI where *I*_max_ was 24% greater in the loaded tibiae than the control tibiae with no rest-insertion versus 16% when a rest insertion was included. However, post-hoc comparisons indicated that despite this statistical interaction, these relative load-induced increases in *I*_max_ were not different. Loading led to increases in Tt.Ar at the 25%, 37% and 50% cortical VOIs as well as increases in Ma.Ar at the 25% VOI, independent of rest insertion (Table 2).

**Table 2:** MicroCT measured parameters of diaphyseal cortical bone at distances of 25%, 37%, 50% and 75% of the tibial length from its proximal end and metaphyseal cancellous bone, in mice subjected to axial compressive loading for 2 weeks under 216 daily load cycles with and without rest insertion.

Parameters		216 Cycles without Rest		216 Cycles with 10 s Rest	
		Control	Loaded	Control	Loaded
Diaphyseal Cortical Bone					
Ct.Ar (mm^2^)	25% ^A^	0.878±0.051	0.981±0.033	0.866±0.038	0.962±0.024
	37% ^A^	0.797±0.035	0.932±0.038	0.791±0.041	0.907±0.033
	50% ^A^	0.632±0.028	0.716±0.032	0.635±0.042	0.707±0.034
	75% ^A^	0.574±0.032	0.582±0.033	0.560±0.023	0.577±0.028
Tt.Ar (mm^2^)	25% ^A^	3.584±0.218	3.904±0.273	3.557±0.354	3.775±0.248
	37% ^A^	2.994±0.181	3.227±0.196	3.076±0.169	3.213±0.195
	50% ^A^	1.508±0.099	1.609±0.091	1.532±0.120	1.628±0.101
	75%	1.153±0.069	1.199±0.128	1.144±0.077	1.172±0.067
Ma.Ar (mm^2^)	25% ^A^	2.706±0.185	2.923±0.259	2.691±0.319	2.813±0.230
	37%	2.197±0.157	2.296±0.169	2.285±0.186	2.306±0.176
	50%	0.877±0.075	0.893±0.070	0.897±0.081	0.921±0.086
	75%	0.580±0.051	0.617±0.138	0.584±0.061	0.595±0.050
Ct.Th (mm)	25% ^A^	0.183±0.005	0.201±0.007	0.180±0.007	0.196±0.006
	37% ^A^	0.190±0.004	0.217±0.005	0.190±0.006	0.213±0.005
	50% ^A^	0.218±0.006	0.244±0.008	0.216±0.007	0.240±0.010
	75% ^A^	0.235±0.009	0.239±0.009	0.231±0.005	0.236±0.010
*I*_max_ (mm^4^)	25% ^A^	0.281±0.034	0.315±0.035	0.282±0.019	0.310±0.026
	37% ^A^	0.217±0.021	0.257±0.028	0.218±0.024	0.249±0.026
	50% ^A^^,^^C^	**0.074±0.009**	**0.092±0.009**	**0.077±0.011**	**0.090±0.011**
	75%	0.051±0.005	0.051±0.005	0.050±0.005	0.051±0.005
*I*_min_ (mm^4^)	25% ^A^	0.120±0.016	0.154±0.020	0.120±0.011	0.150±0.013
	37% ^A^	0.078±0.008	0.101±0.009	0.079±0.009	0.098±0.008
	50% ^A^	0.062±0.006	0.069±0.007	0.063±0.009	0.069±0.008
	75% ^A^	0.040±0.004	0.041±0.005	0.040±0.004	0.041±0.004
Ct.TMD (mg HA/cm^3^)	25%	1033±6	1033±8	1035±7	1033±9
	37% ^A^	1050±9	1043±8	1053±6	1042±8
	50%	1113±9	1118±8	1112±7	1114±7
	75%	1101±9	1103±8	1098±6	1100±10
Metaphyseal Cancellous Bone					
TV (mm^3^)		1.83±0.14	1.87±0.15	1.86±0.11	1.82±0.27
Tb.N (1/mm)		3.6±0.2	3.6±0.3	3.7±0.2	3.5±0.3
Tb.Sp (μm)		274±20	273±25	264±14	280±30
Cn.TMD (mg HA/cm^3^) ^A^		759±9	786±9	762±18	778±13

Data are given as mean ± SD.

^A^ main effect of loading;

^B^ main effect of rest insertion;

^C^ interactive effect of loading and rest insertion (within-subject factor: control vs. loaded, between-subject factor: 10 s rest vs. no rest).

Bold denotes a difference between loaded and control tibiae within each loading group when an interaction is present.

**Fig 6:**
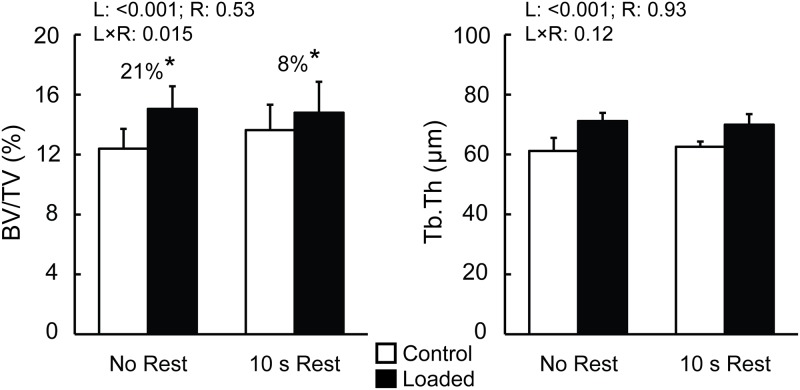
Effect of rest insertion on bone volume fraction (BV/TV) and trabecular thickness (Tb.Th) in the proximal metaphyseal cancellous bone. * indicates significant difference between the control and loaded tibiae within the respective loading group. P values indicating main effect of loading (L), main effect of rest insertion (R) and their interaction (L×R) are listed on top left of each plot. Percent differences were calculated as: (loaded−control) ⁄control×100.

In the metaphyseal cancellous VOI, BV/TV was 8% and 21% greater in the loaded relative to control tibiae loaded under 216 load cycles with and without inserting a rest interval, respectively (Fig 6, S2 Table). While both of these load-induced increases were significant, they did not actually differ from each other, as shown by post-hoc comparison analyses (Fig 6, S2 Table). Tb.Th and Cn.TMD showed similar increases in response to loading with and without a rest insertion (Table 2 and S2 Table). Tb.N and Tb.Sp were not affected by load regardless of rest insertion. Cancellous TV was identical for control and loaded limbs across all groups and was unaffected by loading (Table 2).

## Discussion

The objective of this study was to investigate the effects of load cycle number and short rest insertions on the osteogenic responses of cancellous and cortical bone within the mouse tibia subjected to in vivo axial compressive loading. Using a loading regimen based upon an interrupted triangle waveform with peak loads of -9 N and loading 5 days per week for 2 weeks, we found that, as few as 36 load cycles per day were able to induce osteogenic responses in both cancellous and cortical bone tissues in the mouse tibia. At the 25%, 37% and 50% cortical bone levels relative to the proximal end of the tibia, cortical bone area and thickness continued to increase through 1200 daily load cycles. In the metaphyseal cancellous bone, trabecular thickness increased with load only up to 216 cycles and did not increase beyond this up to 1200 cycles. We also found that insertion of a 10 s rest after every fourth load cycle did not improve the osteogenic response of the cortical or cancellous tissues compared to continuous loading with the same number of load cycles. These results suggest that the load-induced osteogenesis of cancellous bone in the mouse tibia saturates faster than cortical bone in response to increasing daily load cycle numbers, and inter-cycle rest insertion provides no additional osteogenic effect for either cancellous or cortical bone in the mouse tibial loading model given the age and sex of the mice and the loading parameters used here.

**Fig 7:**
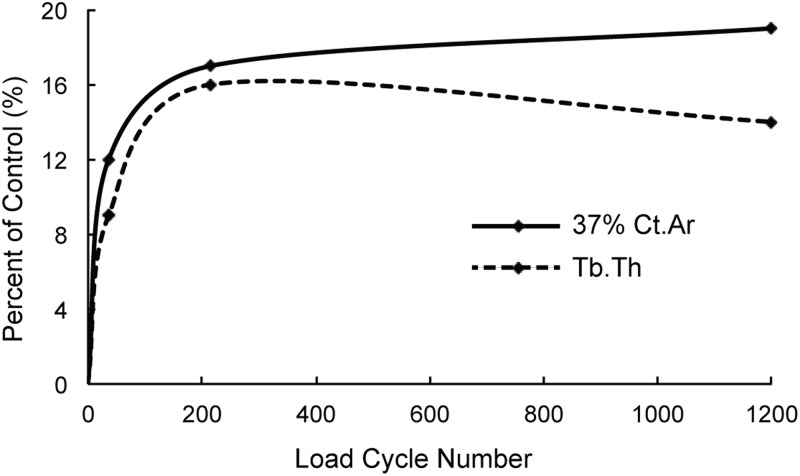
The load-induced increases (percent of control) in cortical bone area (Ct.Ar) of the 37% cortical VOI and trabecular thickness (Tb.Th) in the metaphyseal cancellous VOI, as a function of load cycle number (36, 216, 1200). Note that the increases in these outcome parameters for zero daily load cycle (no load is applied) are assumed as zero. The trend lines presented are best-fit lines. Similar trends were observed for Ct.Th and *I*_min_ of the 37% cortical VOI, Ct.Ar, Ct.Th and *I*_min_ of the 25% and 50% cortical VOIs. Refer to Figs 3 and 5 and Table 1 for detailed statistical results and standard deviations.

Our results that relatively few load cycles (i.e. 36) are sufficient to induce osteogenic responses in cortical and cancellous bone are consistent with previous findings for cortical bone from avian and rodent applied loading studies [4–6, 35]. Furthermore, our results demonstrate that prolonged daily loading (i.e. 1200 or 216 cycles) can enhance the cortical and cancellous bone responses to loading. This result is not entirely consistent with previous observations from the isolated avian ulna loading model showing that 36 cycles of daily loading were just as effective in promoting cortical bone formation as 1800 daily load cycles [4]. Inconsistency between that study and ours could be attributed to the non-physiological strain distribution engendered in the ulnar cortical bone by the applied loads [4] versus a relatively physiological strain distribution produced in the cortical bone in the mouse tibial loading model [12, 14]. Bone cells appear to become accustomed to habitual strain environments induced during daily locomotor loading and abnormal strains engendered during unusual loading situations tend to induce intense adaptive responses [7]. Therefore, fewer load cycles of abnormal strains may produce significant osteogenic responses as effectively as numerous load cycles that induce bone strains that are more physiological in nature [36]. Our current results for the mouse tibial loading model suggest that a longer loading duration may be required to achieve a *maximal* osteogenic response for a physiological loading condition. These results are in agreement with the observations from a rat jump study, which showed that 100 jumps/day induced a statistically greater response in cortical bone compared to 5–40 jumps/day [6]. In that study, a relatively physiological strain environment was presumably generated during jumping. Despite our results showing that prolonged daily loading (e.g. 1200 cycles) can significantly enhance the osteogenic response of the cortical bone compared to short-duration daily loading (e.g. 36 cycles), these results also do not entirely refute the concept of bone cell saturation in response to increasing load cycles. As the number of load cycles applied here increased, load-induced changes in geometric measures of cortical bone did not increase proportionally; instead, they tended to approach a horizontal asymptote (Fig 7). This observation is completely consistent with prior findings showing that there is a diminished return in load-induced osteogenesis in response to increasing load cycle number [2, 7, 37].

While the cortical bone response to loading increases all the way through 1200 cycles, the osteogenic response of the cancellous bone does not increase beyond 216 cycles. This result suggests that cancellous bone’s osteogenic response appears to reach a full saturation faster than the cortical bone. The difference between cortical and cancellous bone responses to increasing loading duration could be explained by the fact that strain magnitudes in the metaphyseal cancellous bone are lower than the diaphyseal cortical bone [17, 28] in the mouse tibia subjected to compressive loading. This would further suggest that the load duration-related response could be strain magnitude dependent, with bone tissues that are subjected to lower peak strains reaching a load duration saturation at fewer load cycles than tissues subjected to greater strain magnitudes. It is also possible that the cellular mechanisms of the cancellous and cortical bone tissues’ responses to mechanical loading could be different [38] due to differences in their biological microenvironment [39, 40]. Another possibility is that longer loading durations (e.g. 1200 cycles) could potentially cause damage to the articular cartilage and subchondral bone [41], which could subsequently affect the osteogenesis of the underlying metaphyseal cancellous bone at a higher number of load cycles. Clearly, further research is required to address this issue.

Several studies using different loading models than the one used here have demonstrated that insertion of short rest intervals (≥ 10 s) between individual load cycles can enhance the osteogenic response for cortical bone [1, 3, 8, 10]. In contrast, we find no benefit of 10 s inter-cycle rest insertions on the osteogenesis of cortical or cancellous bone. These contradictory results could be attributable to the inherent differences between different animal loading models. For the cantilever-loading model, the tibia is loaded in cantilever bending about the anterior-posterior axis with the medial surface in compression and the lateral surface in tension [42]. For the mouse tibial compressive loading model used in this study, loads are applied to the tibia through knee and ankle joints resulting in compression in the posterior aspects and tension in the anterior aspects of the tibia due to its natural curvature [28]. Furthermore, in the prior studies, the strain magnitudes engendered in the cortical bone regions of interest by the applied loads were relatively low, with the peak strains ranging from about -700 με to -1600 με [1, 3, 8, 10]. In the current study, a peak force of -9 N would induce 1780 με at the strain gauge position on the medial surface of the tibial midshaft, corresponding to peak compressive strains of about -3780 με and -3400 με in the mid-shaft cortical bone and metaphyseal cancellous bone within the mouse tibia, respectively, based upon our previous study using finite element analysis combined with strain gauge measures [28]. These results may suggest that the osteogenic effect of short rest insertions may be suppressed by high-magnitude loads or strains. This hypothesis is consistent with the results reported by another study using the mouse tibia cantilever-bending model, which demonstrated that inserting 10 s or 20 s rests did not have any effect on additionally enhancing cortical bone formation resulting from high-magnitude loads (corresponding to about -2400 με peak strain at the tibial midshaft), whereas 10 s or 20 s rest insertions did improve cortical osteogenesis for low-magnitude loads (corresponding to about -1200 με peak strain at the tibial midshaft) [32].

In addition to the possibilities mentioned above, the difference in experimental design for studying the effect of rest insertion could potentially be another factor. In previous mouse cantilever bending studies [3, 8], the total loading time was held constant and the number of load cycles varied in order to compensate for the added rest periods. In the current study, the total number of load cycles was held constant resulting in varied total loading times, similar to several other studies that have examined the influence of rest insertion on load-induced osteogenesis [1, 10, 32]. We chose to maintain the total number of loading cycles constant in order to test the effect of only rest insertion, rather than the effects of changing both rest insertion and the total number of load cycles. The question then arises whether or not the increase in total loading time as a result of inserting 10 s rest phases would have a negative effect on further enhancing load-induced osteogenesis. By keeping the number of load cycles the same and varying only the length of rest time (and thus the total loading time), Robling et al [1] used the rat four-point bending model and found that insertion of 14 s rest periods significantly increased the osteogenic response compared to continuous loading and other shorter rest-inserted loading regimes (3.5 s and 7 s). In that study, the total loading time of the 14 s rest group (8 min) is about 27 times longer than the group with no rest insertion applied (18 s). This result may suggest that the increase in the total loading time as a result of insertion of rest periods might not exhaust the loading response, although this result was found using the rat four-point bending model and not the tibial loading model that we used here. Another difference in our experimental design compared to some other studies examining rest insertion is that rests were inserted between every four load cycles in this study while a rest was inserted following every cycle in some other studies [1, 3, 32]. Furthermore, it remains a possibility that the 0.1 s short dwells included following each loading cycle in all four loading protocols may have diminished the osteogenic potential of the 10 s rests. While this very short inter-cycle rest seems negligible, its effects compared to a true ‘sawtooth’ triangular waveform remain unknown and may require further studies.

The number of load cycles tested here (36, 216, 1200) represented several typical values that have been commonly used in previous loading studies [3, 4, 11, 17, 25, 26]. We did not attempt to test any number of load cycles below 36. A previous study using the mouse tibia cantilever-bending model found that 50 cycles of continuous loading per day was osteogenic whereas 10 cycles per day was not, given the loading parameters that they used [10]. A rat jumping study showed that 5 jumps per day were osteogenic [6]. It remains unclear what minimum cycle number is necessary to induce bone formation in the mouse axial compression tibial loading model. However, 36 load cycles using our waveform only takes 9 s and even lower cycle numbers would likely provide little advantage over 36 cycles in terms of total experimental time and minimization of discomfort to mice. We did not attempt to test any cycle number above 1200 since that would potentially cause articular cartilage and subchondral bone trauma in the knee joint in this model [41], which could subsequently affect bone’s anabolic response. Furthermore, a clear saturation trend could be observed when the load cycle number increased from 36 to 1200 (Fig 7), suggesting further enhancement by increasing the load cycle number beyond 1200 would likely be minimal.

In addition to the mechanistic insights provided by our study, the current findings may also be useful in providing comparative results and useful guides for conducting future mouse tibial loading studies. Since our results show that very few daily load cycles (i.e. 36) are sufficient to generate bone formation in both cancellous and cortical bone and about 216 cycles can produce relatively large cortical and maximal cancellous responses, mechanistic studies examining load-induced cancellous and cortical bone formation could be accomplished with greater throughput with relatively few load cycles. Fewer daily load cycles could provide the additional benefit of possibly reducing load-induced soft tissue trauma in the knee [41]. Furthermore, the loading and anesthesia time could be further reduced by excluding short rest intervals when using the mouse tibial loading model. It appears that 1200 cycles of daily loading may be associated with some woven bone formation at the lateral periosteal surface at the 37% cortical VOI, based upon microCT scans (Fig 4). This would further support a recommendation for using a lower number of load cycles for tibial loading studies, if formation of woven bone would be an undesirable experimental outcome.

A few limitations to this study need to be considered when generalizing the current results. First, the effects of strain magnitude, strain rate, cycle frequency and alternative loading waveforms were not investigated in this study. The loading parameters used here were adopted from a commonly used loading protocol for the mouse tibial loading model [11, 17, 26, 27] and maintained the same for all four loading protocols. Different research groups also use different waveforms applied at different frequencies over a varying number of days than used here [1, 15, 31, 43, 44]. Regardless, our results showing the relative effects of increasing load cycle number should be maintained despite varying some of the other parameters. Second, our results only focused on the load-induced changes in bone mass and architecture of the cortical and cancellous bone over a period of two weeks, as measured by microCT. We did not specifically measure the effects of the various loading protocols on bone formation rate by 2D histomorphometry. For our analyses of cancellous bone, 2D histomorphometry would possibly not add any additional useful information given the relatively low BV/TV and irregular and varying cancellous architecture in the proximal tibia, which produces a spatially-variant strain environment in these tissues [28]. Due to these factors, the results of any 2D histomorphometric analysis for the proximal tibia will be heavily dependent upon where the sections are taken for the control and loaded limbs, which can be difficult to control given the small size of the mouse proximal tibia [45]. It has previously been shown that volumetric microCT analyses may be more sensitive for measuring load-induced changes in the cancellous compartment in the proximal tibia than 2D dynamic histomorphometry for tibial loading studies, since dynamic histomorphometry analyses may not reproduce the load effect results indicated by volumetric microCT analyses [17, 20]. We also did not perform in vivo microCT in order to monitor morphometric and tissue mineral density changes in the same bone over the course of the loading experiment. Repeated in vivo scanning may not have been feasible for this study for several reasons. Firstly, this study was designed to analyze load-induced responses in multiple cortical VOIs located along the entire tibia (25%, 37%, 50% and 75% relative to the proximal end) as well as the proximal cancellous VOI. It took about 3.5 hrs to scan a whole tibia ex vivo using our settings, which are similar to settings used for in vivo microCT scanning [45]. For in vivo microCT scanning, such a long scanning (and anesthesia) time could be harmful to mice and potentially affect the anabolic response of bone tissue to loading. Also, the effect of long-term x-ray radiation on bone tissue may be another concern [17]. Regardless, ex vivo microCT-based evaluation of load-induced responses by comparing the control and loaded tibiae is valid and used commonly.

In summary, we find using the mouse tibial loading model that as few as 36 daily load cycles are sufficient to produce an appreciable osteogenic response in both cancellous and cortical bone with a maximal cancellous response achieved with 216 daily cycles. Osteogenesis of cancellous bone appears to saturate faster than cortical bone in response to increasing load cycle numbers in the mouse tibial loading model with the age and sex of the mice and the loading parameters used here. We also find that inter-cycle rest insertion provides no additional osteogenic effect on cancellous or cortical bone using the loading parameters applied here. These results suggest that relatively few daily load cycles without short rest insertions may be sufficient for using the mouse tibial loading model to study cancellous and cortical bone mechanobiology.

## Supporting Information

S1 Table MicroCT measured parameters of diaphyseal cortical bone at distances of 25%, 37%, 50% and 75% of the tibial length from its proximal end and metaphyseal cancellous bone, in mice subjected to axial compressive loading for 2 weeks under different daily load cycles (36, 216, 1200). (DOC)

S2 Table MicroCT measured parameters of the metaphyseal cancellous bone, in mice subjected to axial compressive loading for 2 weeks under 216 daily load cycles with and without rest insertion. (DOC)
