# Supplementary material for: Effects of Loading Duration and Short Rest Insertion on Cancellous and Cortical Bone Adaptation in the Mouse Tibia
Source: PLoS One. 2017 Jan 11;12(1):e0169519. doi: 10.1371/journal.pone.0169519 (PMC5226737; doi:10.1371/journal.pone.0169519)
Supplement: S1 Table — (DOC) [file pone.0169519.s001.doc]

**S1 Table** MicroCT measured parameters of diaphyseal cortical bone at distances of 25%, 37%, 50% and 75% of the tibial length from its proximal end and metaphyseal cancellous bone, in mice subjected to axial compressive loading for 2 weeks under different daily load cycles (36, 216, 1200).

| Parameters | | 36 Cycles | | |  | | 216 Cycles | | |  | | 1200 Cycles | | | |
| --- | --- | --- | --- | --- | --- | --- | --- | --- | --- | --- | --- | --- | --- | --- | --- |
|  |  | Control | | Loaded |  |  | Control | | Loaded |  |  | Control | | | Loaded |
| Diaphyseal Cortical Bone | | | |  | | |  | |  | |  | | | |  |
| Ct.Ar (mm^2^) | 25% ^A,B,C^ | **0.899±0.061** | **0.976±0.050** | | | **0.878±0.051** | | **0.981±0.033** | | | | | **0.908±0.039** | **1.052±0.052** ^d,e^ | |
|  | 37% ^A,B,C^ | **0.810±0.039** | **0.907±0.023** | | | **0.797±0.035** | | **0.932±0.038** | | | | | **0.832±0.026** | **0.987±0.027** ^d,e^ | |
|  | 50% ^A,B,C^ | **0.651±0.027** | **0.712±0.028** | | | **0.632±0.028** | | **0.716±0.032** | | | | | **0.664±0.018** | **0.762±0.033** ^d,e^ | |
|  | 75% ^A^ | 0.586±0.025 | 0.595±0.023 | | | 0.574±0.032 | | 0.582±0.033 | | | | | 0.594±0.029 | 0.596±0.029 | |
|  |  |  |  | | |  | |  | | | | |  |  | |
| *I*_min_ (mm^4^) | 25% ^A,C^ | **0.133±0.024** | **0.152±0.021** | | | **0.120±0.016** | | **0.154±0.020** | | | | | **0.133±0.015** | **0.174±0.025** | |
|  | 37% ^A,B,C^ | **0.083±0.009** | **0.099±0.010** | | | **0.078±0.008** | | **0.101±0.009** | | | | | **0.085±0.006** | **0.113±0.007** ^d,e^ | |
|  | 50% ^A,B^ | 0.068±0.007 | 0.074±0.009 | | | 0.062±0.006 | | 0.069±0.007 | | | | | 0.071±0.004 | 0.079±0.004 | |
|  | 75% ^A^ | 0.042±0.003 | 0.043±0.003 | | | 0.040±0.004 | | 0.041±0.005 | | | | | 0.044±0.003 | 0.044±0.003 | |
| Metaphyseal Cancellous Bone | | | |  | | |  | |  | |  | | | |  |
| BV/TV (%) ^A,B^ | | 11.9±1.3 | 13.5±1.5 | | | 12.4±1.7 | | 15.0±2.1 | | | | | 13.9±1.5 | 15.6±2.0 | |
| Tb.Th (µm) ^A,B,C^ | | **61±4** | **67±3** | | | **61±2** | | **71±3** ^d^ | | | | | **64±3** | **74±4** ^d^ | |

Data are given as mean ± SD.

^A^ main effect of loading; ^B^ main effect of load cycle number; ^C^ interactive effect of loading and load cycle number (within-subject factor: control vs. loaded, between-subject factor: number of load cycles applied).

^d^ different from the loaded tibiae for 36 cycles (no difference between nonloaded controls); ^e^ different from the loaded tibiae for 216 cycles (no difference between nonloaded controls).

Bold denotes a difference between the loaded and control tibiae within each load cycle group when an interaction is present.
