# Supplementary material for: Effects of Loading Duration and Short Rest Insertion on Cancellous and Cortical Bone Adaptation in the Mouse Tibia
Source: PLoS One. 2017 Jan 11;12(1):e0169519. doi: 10.1371/journal.pone.0169519 (PMC5226737; doi:10.1371/journal.pone.0169519)
Supplement: S2 Table — (DOC) [file pone.0169519.s002.doc]

**S2 Table MicroCT measured parameters of the metaphyseal cancellous bone, in mice subjected to axial compressive loading for 2 weeks under 216 daily load cycles with and without rest insertion.**

| Parameters | 216 Cycles without Rest | |  | 216 Cycles with 10 s Rest | |
| --- | --- | --- | --- | --- | --- |
|  | Control | Loaded |  | Control | Loaded |
| Metaphyseal Cancellous Bone | |  | |  |  |
| BV/TV (%) ^A,C^ | **12.4±1.7** | **15.0±2.1** | | **13.6±1.7** | **14.8±1.8** |
| Tb.Th (µm) ^A^ | 61±2 | 71±3 | | 63±4 | 70±4 |

Data are given as mean ± SD.

^A^ main effect of loading; ^B^ main effect of rest insertion; ^C^ interactive effect of loading and rest insertion (within-subject factor: control vs. loaded, between-subject factor: 10 s rest vs. no rest).

Bold denotes a difference between loaded and control tibiae within each loading group when an interaction is present.
